# Effects of Biochar- and Hydrochar-Amended Organic Fertilizer on Crop Production, NH_3_ Loss, and Fertility of Coastal Saline–Alkali Soil

**DOI:** 10.3390/plants14233616

**Published:** 2025-11-27

**Authors:** Chang Liu, Wang Huang, Yanfang Feng, Meng Ma, Haijun Sun

**Affiliations:** 1 Co-Innovation Center for Sustainable Forestry in Southern China, College of Forestry and Grassland, Nanjing Forestry University, Nanjing 210037, China; liuchang@njfu.edu.cn (C.L.); huangwang@njfu.edu.cn (W.H.); mameng@njfu.edu.cn (M.M.); 2 National Positional Observatory for the Changjiang River Delta Farmland Protection Forest Ecosystem, Jiangsu Province, State Forestry and Grassland Administration, Nanjing 210037, China; 3 Institute of Agricultural Resources and Environment, Jiangsu Academy of Agricultural Sciences, Nanjing 210014, China; yfeng@jaas.ac.cn

**Keywords:** biochar, N fertilizer, NH_3_ volatilization, rice–wheat rotation, soil nutrient

## Abstract

Biochar- and hydrochar-amended organic fertilizers are widely used to enhance saline–alkali soil fertility and crop production. However, their effects on ammonia (NH_3_) volatilization from saline–alkali soils remain unclear. Here, we conducted a pot experiment to investigate the impacts of organic fertilizer (OF), and of that with biochar (BC-OF) and hydrochar (HC-OF) amendments at a rate of 15% on crop production, on soil fertility and NH_3_ loss in saline–alkali soil with rice–wheat rotation, using chemical fertilizer alone as the control (CK). Compared with CK, OF, BC-OF, and HC-OF increased the rice and wheat yields. In particular, HC-OF harvested a significant 16.6% more grain yield than OF (*p* < 0.05). Organic fertilizer amendments exerted a general mitigating effect on the NH_3_ volatilization during different observations after nitrogen (N) fertilization. Correspondingly, they reduced total NH_3_ volatilization during the rice season compared to CK. Compared with CK, HC-OF significantly reduced NH_3_ emissions by 61.6% during the wheat season and 28.3% annually (*p* < 0.05). Moreover, HC-OF treatment reduced total NH_3_ volatilization in the wheat season by 55.8% and 64.7% compared to OF and BC-OF, respectively. Compared with CK, HC-OF treatment significantly reduced soil pH, while both the BC-OF and HC-OF treatments increased soil total N and ammonium N contents, even surpassing those in the OF treatment. However, no significant differences were observed among treatments in soil electrical conductivity, nitrate, available phosphorus and potassium, as well as organic matter content. In conclusion, HC-OF is more suitable for enhancing crop yield and reducing soil N loss in saline–alkali soils.

## 1. Introduction

As a typical degraded soil type, saline–alkali soil has low agricultural productivity due to its high salinity and alkalinity, low nutrient availability, and poor physical structure [1,2]. The area of salt-affected farmland in China has reached 8.2 × 10^7^ hm^2^, accounting for approximately 6.2% of the total arable land [3]. This critical situation underscores the remediation of saline–alkali soils as a strategic imperative for ensuring national food security and safeguarding arable land resources [4]. Currently, the paddy–upland rotation system represents a predominant agricultural practice for saline soil reclamation, which can enhance grain yield in the short-term [5]. However, long-term intensive cultivation leads to alterations in soil organic matter and nutrient content within the tillage layer. Furthermore, the excessive saline ions in saline–alkali soils impose substantial osmotic and ionic stress on the soil, resulting in the deterioration of soil physicochemical properties, structural degradation and a persistent decline in soil quality [6]. Therefore, improving saline–alkali soils is of great significance for enhancing land-use efficiency and crop productivity.

Application of organic fertilizers is an effective measure for improving soil physicochemical properties and enhancing crop productivity [7]. Biochar-amended organic fertilizer, a novel organic amendment, is primarily produced from biomass-derived biochar and formulated with waste materials from the livestock industry [8]. One previous work indicated that biochar-amended organic fertilizers are rich in organic matter, which can promote crop growth and development by improving soil structure, enhancing microbial activity, and providing slow-release nutrients [9]. Moreover, biochar-amended organic fertilizers possess notable properties such as high porosity, strong adsorption capacity, and stability, making them widely applicable in soil amendment. For instance, biochar-amended organic fertilizers contain abundant surface functional groups, which facilitate nutrient retention in the rhizosphere soil, thereby increasing nutrient availability [10]. Research has also demonstrated that biochar-amended organic fertilizers can significantly improve soil physical structure and biochemical properties, leading to enhanced soil productivity, greater dry matter accumulation and increased crop yield [11,12]. Similarly, hydrochar-amended organic fertilizers have been proven to be an effective soil amendment [13], improving soil health, boosting productivity, and supporting sustainability through moisture retention, nutrient supply, and microbial activity stimulation [14]. Additionally, the surface of hydrochar contains acidic functional groups, which may help neutralize high pH levels in alkaline soils, thereby increasing nutrient solubility and availability to promote plant growth [15]. However, the efficacy of organic fertilizers in soil improvement varies depending on factors such as crop type, feedstock composition, carbonization process, regional climate conditions, and soil texture. Furthermore, compared to conventional chemical fertilizers, organic fertilizers generally contain a lower content of readily available nutrients (e.g., nitrogen and phosphorus) and exhibit slower release rates [16]. Therefore, during critical growth stages of crops, the appropriate application of organic fertilizers that amended with biochar or hydrochar should be considered to provide multiple benefits for soil enhancement and yield improvement [17].

Excessive and irrational fertilizer application in agricultural production has become one of the major contributors to non-point source pollution and ecological degradation, with nitrogen (N) losses being particularly prominent [18]. Ammonia (NH_3_) volatilization represents a primary pathway of N loss from croplands, accounting for 10–60% of applied N fertilizers [19]. Its emission intensity is significantly influenced by soil physicochemical properties and environmental conditions. In saline–alkali soils, in particular, elevated pH and salinity inhibit plant N uptake, while chloride ions impede NO_3_^−^-N absorption. Concurrently, high salinity increases NH_4_^+^-N concentration and reduces nitrification rates, thereby exacerbating NH_3_ volatilization losses [20,21]. In recent years, biochar-amended organic fertilizers have garnered considerable attention due to their regulatory effects on soil N cycling. Research indicates that biochar-amended organic fertilizers can influence NH_3_ emissions through multiple mechanisms. On the one hand, the surfaces of biochar-amended organic fertilizers are typically enriched with functional groups (e.g., amino and carboxyl groups), which can adsorb NH_4_^+^, thereby mitigating NH_3_ volatilization from agricultural soils [22]. On the other hand, biochar-amended organic fertilizers may indirectly increase NH_3_ emissions by altering soil properties such as pH and electrical conductivity (EC) [23], an effect particularly pronounced in saline–alkali soils characterized by high pH and salinity. However, compared to conventional arable soils, saline–alkali soils exhibit distinct differences in physical structure, chemical properties, and microbial activity. To date, research on the dynamic changes in soil–water properties and nutrient cycling within saline–alkali soil–crop systems following biochar-amended organic fertilizer application remains limited. Furthermore, NH_3_ volatilization dynamics under rice–wheat rotation systems in saline–alkali soils are still unclear, which hinders the optimized application of biochar-amended organic fertilizers in saline–alkali agriculture.

Although biochar-amended organic fertilizer serves as an important alternative to chemical N fertilizer for improving soil nutrient availability, its effects on soil fertility characteristics and NH_3_ emission dynamics remain insufficiently elucidated. Therefore, we conducted a one-year rice–wheat rotation pot experiment to investigate the influence of biochar- and hydrochar-amended organic fertilizer application on NH_3_ emissions and soil amelioration in saline–alkali soils. We compared organic fertilizer (OF), biochar-amended organic fertilizer (BC-OF), and hydrochar-amended organic fertilizer (HC-OF) to assess their effects on soil nutrient dynamics and NH_3_ volatilization dynamics. We hypothesized that these two organic fertilizer applications would significantly mitigate NH_3_ emissions, enhance crop productivity and sustain long-term soil fertility. The objectives of the study were to (1) evaluate the crop yield response to biochar- and hydrochar-amended organic fertilizer treatments in saline–alkali soils; (2) determine whether these two organic fertilizers improve soil physicochemical properties and nutrient retention capacity; and (3) elucidate the effects of the aforementioned organic fertilizers on NH_3_ emission dynamics of saline–alkali soil.

## 2. Results

### 2.1. Yield and Agronomic Traits of Rice and Wheat

Compared with the CK treatment, the three organic fertilizers increased grain yields of rice and wheat to varying degrees. Among them, the HC-OF treatment significantly increased wheat grain yield by 18.6% and 16.6% compared to the CK and OF treatments, respectively (Table 1) (*p* < 0.05). This is likely attributable to the increased number of panicles and grains per panicle under the HC-OF treatment. The thousand-kernel weight of rice and wheat showed slight decreases following application of organic fertilizers. Both biochar- and hydrochar-amended organic fertilizer treatments increased annual grain yield, with HC-OF achieving the highest yield (122.45 g/pot), representing a 3.6% increase over CK.

### 2.2. N Utilization Efficiency in Rice and Wheat

**Figure 1:**
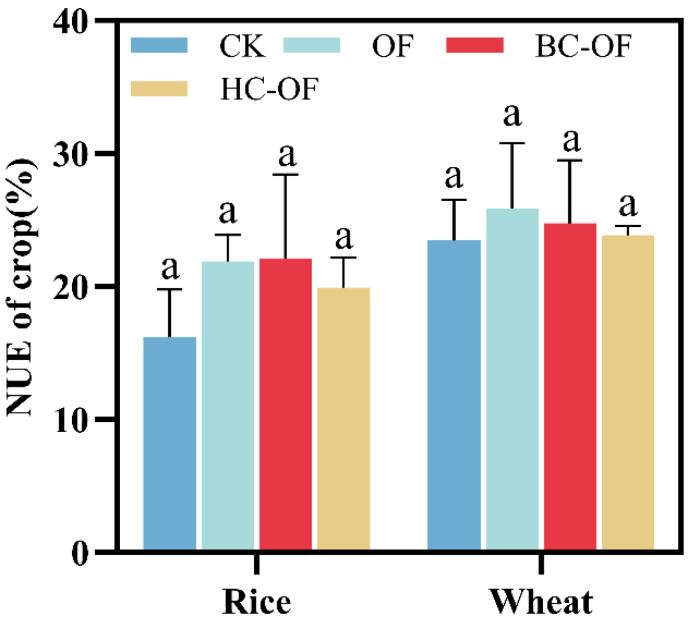
Nitrogen utilization efficiency (NUE) of rice and wheat planted under control (CK), organic fertilizer (OF), biochar-amended organic fertilizer (BC-OF), and hydrochar-amended organic fertilizer (HC-OF) treatments. Data represent means ± SD (*n* = 4). Different lowercase letters above bars indicate significant differences (*p* < 0.05) according to Duncan’s multiple range test.

Compared to the CK treatment, all three organic fertilizer treatments exhibited increased nitrogen utilization efficiency (NUE) in both rice and wheat, with the enhancement ranging from 1.8% to 36.6% (Figure 1). The BC-OF treatment showed the highest NUE in the rice season, whereas the OF treatment achieved the highest NUE in the wheat season, though no significant differences were observed among the treatments.

### 2.3. NH_3_ Volatilization Analysis

**Figure 2:**
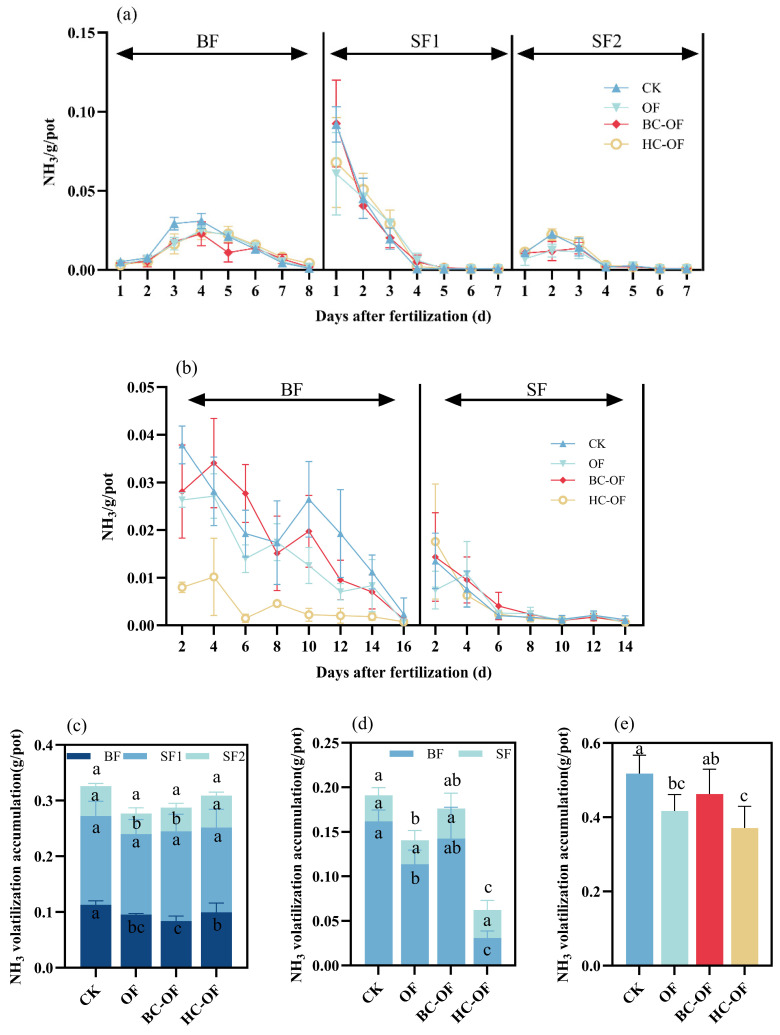
Effects of organic fertilizer (OF) and biochar- (BC-OF) and hydrochar-amended organic fertilizer (HC-OF) on NH_3_ volatilization in saline–alkali soil. (**a**) Dynamic NH_3_ emission fluxes during the rice season. (**b**) Dynamic NH_3_ emission fluxes during wheat season. (**c**) Cumulative NH_3_ volatilization in rice season. (**d**) Cumulative NH_3_ volatilization in wheat season. (**e**) Annual scale NH_3_ losses. BF, basal fertilizer stage; SF: supplemental fertilization stage; SF1, tillering fertilization stage; SF2, panicle fertilization stage. Data represent means ± SD (*n* = 4). Different lowercase letters above bars indicate significant differences (*p* < 0.05) according to Duncan’s multiple range test.

During the tillering fertilization stage (SF1), the observed NH_3_ volatilization flux in the rice season peaked at 1 day after fertilization and subsequently showed a continuous decreasing trend (Figure 2a). NH_3_ volatilization from all treatments peaked during days 2–3 after fertilization in both basal fertilizer stage (BF) and panicle fertilization (SF2) periods, showing a consistent pattern of initial increase followed by gradual decline. Overall, the observed NH_3_ fluxes during BF and SF2 were lower than those in SF1 across all treatments. Within the first three days of BF in the wheat season, NH_3_ emissions peaked in all treatments before decreasing to varying degrees and showed a slight increase after the sixth day (Figure 2b). At the supplemental fertilization stage (SF), except for OF, all treatments reached peak NH_3_ emissions on the first day after fertilization, with HC-OF exhibiting the highest peak value, followed by a continuous decline. Overall, the total NH_3_ volatilization observed during SF was lower than that during BF.

Throughout the rice and wheat seasons, CK treatment exhibited higher cumulative NH_3_ emissions, while all organic fertilizer applications demonstrated reduced total NH_3_ volatilization. Compared to CK, total NH_3_ volatilization in the rice season was significantly reduced by 15.5% (OF), 25.8% (BC-OF), and 11.9% (HC-OF) during the BF period, and by 31.9% (OF) and 22.4% (BC-OF) during the SF2 period (Figure 2c) (*p* < 0.05). During the wheat season, HC-OF demonstrated the most significant mitigation effect, reducing total NH_3_ emissions by 61.6%, 55.8%, and 64.7% compared to CK, OF, and BC-OF, respectively (Figure 2d) (*p* < 0.05). Across the rice–wheat annual cycle, all organic fertilizer treatments reduced total NH_3_ emissions. The HC-OF treatment produced the greatest mitigation, with cumulative emissions 28.3% and 19.8% lower than those from the CK and BC-OF treatments, respectively.

### 2.4. Floodwater pH, NH_4_^+^-N, and NO_3_^−^-N Concentration in the Rice Season

**Figure 3:**
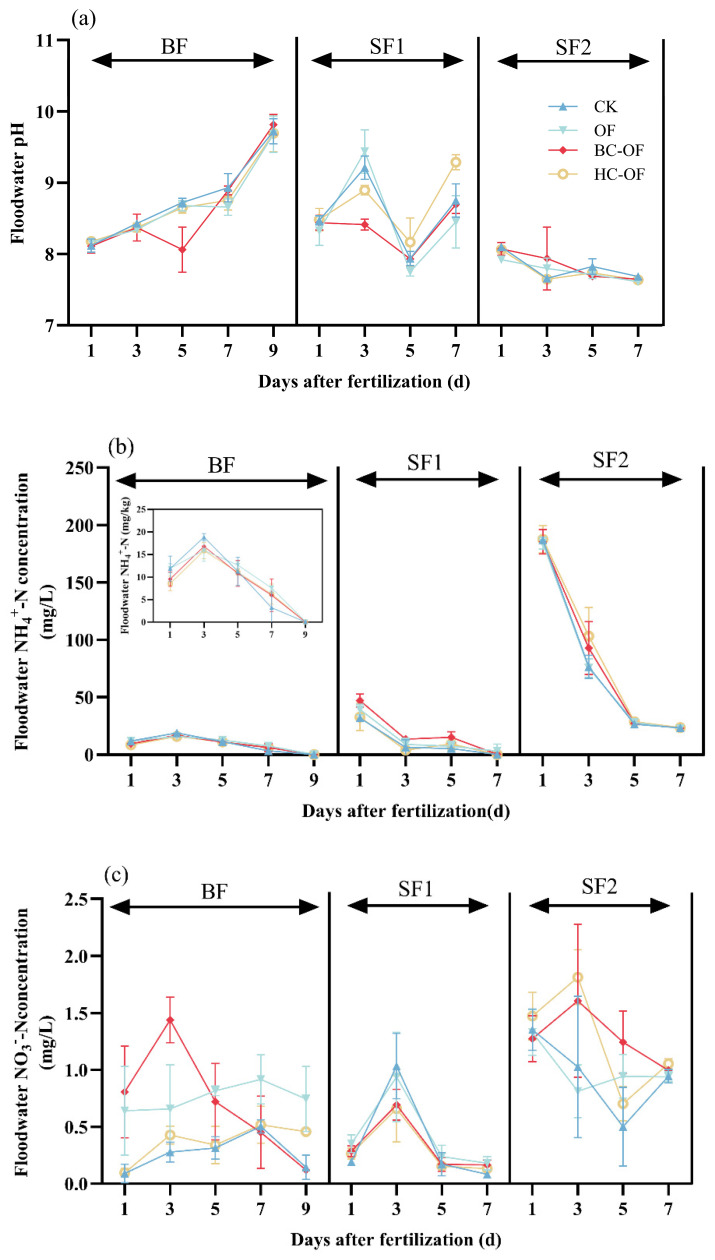
Dynamic pH (**a**), NH_4_^+^-N (**b**), and NO_3_^−^-N (**c**) concentrations of floodwater under organic fertilizer (OF) and biochar- (BC-OF) and hydrochar-amended organic fertilizer (HC-OF) in basal fertilization (BF), first supplementary fertilization (SF1), and second supplementary fertilization (SF2), respectively.

The floodwater pH dynamics exhibited distinct trends across different fertilization stages (Figure 3a). During the BF period, the pH of floodwater exhibited a consistent upward trend, except for the HC-OF treatment which decreased to its lowest value on Day 5. In the SF1 period, the pH values of all treatments generally followed an initial increase followed by a decrease, with a sharp rise observed on the final day of fertilization. Throughout the SF2 observation period, no significant fluctuations in floodwater pH were detected, with only a slight gradual decline being observed.

At BF, floodwater NH_4_^+^-N concentrations in all treatments exhibited a characteristic pattern of initial increase peaking on Day 3 before subsequent decline (Figure 3b). At SF1, all treatments showing rapid NH_4_^+^-N depletion during Days 1–3 were followed by stabilized fluctuations, where BC-OF emerged with the highest residual concentrations among the three organic fertilizer applied treatments. During the SF2, an immediate concentration drop occurred on Day 1 across treatments, stabilizing by Day 5, with HC-OF unexpectedly showing the highest terminal NH_4_^+^-N levels.

Floodwater NO_3_^−^-N concentrations remained relatively low across all treatments, with minimal overall variation (Figure 3c). During the BF, floodwater NO_3_^−^-N concentrations generally exhibited an initial increase followed by a gradual decline. The BC-OF treatment exhibited a sharp increase in NO_3_^−^-N concentration after the first day, peaking on Day 3. In the SF1, NO_3_^−^-N concentrations in all treatments followed a rise-and-fall trend, reaching their peaks on Day 3. During SF2, BC-OF and HC-OF displayed minor peaks on Day 3 before declining, whereas CK and OF exhibited an immediate decreasing trend from Day 1. Notably, after declining until Day 5, CK, OF, and HC-OF experienced a slight rebound in NO_3_^−^-N levels.

### 2.5. Topsoil pH, NH_4_^+^-N, and NO_3_^−^-N Concentration in the Wheat Season

**Figure 4:**
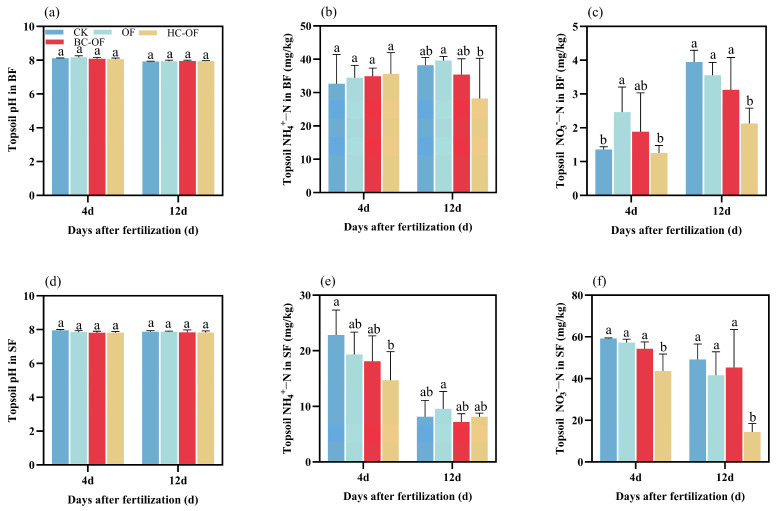
Topsoil pH, NH_4_^+^-N, and NO_3_^−^-N contents of organic fertilizer (OF) and biochar- (BC-OF) and hydrochar-amended organic fertilizer (HC-OF) across different fertilization stages during wheat season. Dynamic pH (**a**,**d**), NH_4_^+^-N (**b**,**e**), and NO_3_^−^-N (**c**,**f**) concentration of topsoil at Day 4 and Day 12 in basal fertilization (BF) and supplementary fertilization (SF), respectively. Data represent means ± SD (*n* = 4). Different lowercase letters above bars indicate significant differences (*p* < 0.05) according to Duncan’s multiple range test.

During both BF and SF, soil pH remained stable across all treatments, showing no significant differences on Days 4 and 12 (Figure 4a,d). In the BF, while soil NH_4_^+^-N content showed no initial variation among treatments during the first 4 days, HC-OF treatment demonstrated significant N retention by Day 12, reducing NH_4_^+^-N content by 28.8% compared to OF (Figure 4b) (*p* < 0.05). The HC-OF treatment reduced NH_4_^+^-N content by 35.6% relative to CK on Day 4 in the SF, with all treatments showing decreased NH_4_^+^-N levels by Day 12 (Figure 4e). During BF, HC-OF treatment reduced NO_3_^−^-N content by 49% compared to OF on Day 4, and by 46.1% and 40.2% relative to CK and OF, respectively (*p* < 0.05) by Day 12 (Figure 4c). During SF, soil NO_3_^−^-N content under HC-OF treatment was consistently lowest at both 4d and 12d, showing significant reductions of 26.4% and 70.6% compared to CK, 23.8% and 65.3% compared with OF, and 19.6% and 68.2% relative to BC-OF (Figure 4f) (*p* < 0.05).

### 2.6. Soil Fertility Characteristics

HC-OF treatment reduced soil pH by 0.13 units relative to CK (Table 2). From the perspective of EC values, the BC-OF treatment exhibited the lowest soil electrical conductivity, but no significant differences were observed among the various organic fertilizer amended treatments. Compared with CK treatment, BC-OF and HC-OF treatments significantly increased soil TN content by 7.5% and 11.3%, respectively (*p* < 0.05). The soil NH_4_^+^-N content in the BC-OF and HC-OF treatments increased significantly by 2.28-fold and 9-fold compared to the CK treatment, respectively, and was even 1.9-fold and 7.8-fold higher than that in the OF treatment (*p* < 0.05). Although all treatments showed elevated NO_3_^−^-N content compared to CK, these differences were not statistically significant. Measurements of AP and AK content showed no significant variations among the three organic fertilizer treatments (OF, BC-OF, HC-OF). Interestingly, HC-OF recorded the highest SOM content (10.30 g/kg) among all treatments, though inter-treatment differences in SOM did not reach statistical significance.

## 3. Discussion

### 3.1. Effects of Different Organic Fertilizers on Rice and Wheat Yield and NUE

For the reclamation and utilization of saline–alkali soils, the application of organic fertilizers is a common agronomic practice [7]. Compared to N fertilizer alone, biochar-amended organic fertilizers provide a balanced supply of both organic and inorganic nutrients, meeting crop demands and sustaining stable yields [24]. In this study, no significant differences in rice grain yield were observed between the BC-OF and HC-OF treatments, though a slight increase was noted (Table 1). Liu et al. [25] found that despite improved soil nutrient availability in rice–wheat systems, biochar-amended organic fertilizers did not significantly enhance rice grain production. However, the HC-OF treatment significantly increased wheat grain yield by 18.6% compared to CK, while BC-OF also exhibited a measurable yield-enhancing effect (Table 1). A rational explanation is that the limited nutrient availability from organic fertilizer during the initial decomposition stage was insufficient to meet the demands of rice growth, resulting in no significant improvement in rice yield [26]. In contrast, biochar-amended organic fertilizer undergoes a longer transformation period, leading to a gradual release of nutrients that enhances the availability of soil N and P during critical growth stages of wheat. This mechanism likely improves fertilizer-use efficiency and ultimately boosts wheat yield [27].

Biochar-amended organic fertilizer application significantly enhances N uptake and use in cereal crops [28]. In this study, compared to CK, both BC-OF and HC-OF demonstrated improved NUE in rice and wheat (Figure 1). Hydrochar-amended organic fertilizer, through microbial aging processes, decomposes readily degradable carbon sources while maintaining its stable carbon skeleton structure, thereby significantly enhancing soil N availability [29]. Our results showed that HC-OF treatment increased soil TN content by 11.3% compared to CK (Table 2). This improvement in N availability promoted crop nitrogen uptake and plant growth, ultimately resulting in the highest yield under HC-OF treatment (Table 1). Additionally, Chew et al. [30] proposed that biochar-induced electrochemical potential gradients in the rhizosphere may drive mineral nutrient absorption, further improving nutrient acquisition. From a yield-maximization perspective, HC-OF exhibited superior performance. However, when considering integrated benefits, including yield, N uptake, and long-term soil amelioration, BC-OF is more suitable for saline–alkali soil remediation and sustainable agricultural production. While having observed crop yield and N use in a short-term pot experiment, future work should involve long-term field studies to evaluate the enduring effects of BC- and HC-OF on both crop yield and quality.

### 3.2. Effects of Different Organic Fertilizers on NH_3_ Volatilization from Saline–Alkali Soil

Current research indicates that the organic fertilizer applications may reduce N losses in agricultural fields by improving soil N retention capacity, including mitigating N leaching and NH_3_ volatilization risks [31]. However, in saline–alkali soils, high pH and soil salinity can promote the conversion of NH_4_^+^ to NH_3_, thereby increasing NH_3_ volatilization [32]. The results of this study demonstrate that during the rice fertilization period, compared to the CK treatment, the application of three organic fertilizers led to varying degrees of reduction in cumulative NH_3_ emissions, with OF and BC-OF treatments exhibiting more pronounced mitigation effects (Figure 2c). Previous studies have also observed that biochar-amended organic fertilizer significantly reduces NH_3_ volatilization in moderately saline soils under incubation experiments [33]. The presence of negatively charged functional groups on the surface of biochar-amended organic fertilizers, which can adsorb NH_4_^+^ in the soil, thereby reducing NH_4_^+^ loss [34]. In this experiment, the HC-OF treatment exhibited significant NH_3_ mitigation effects during the wheat season and the annual rotation cycle, reducing emissions by 28.3% and 61.6% compared to CK (Figure 2d,e). HC-OF possesses a highly aromatic structure and abundant oxygen-containing functional groups, particularly a high density of carboxyl groups, which enhance NH_4_^+^ adsorption and thus partially suppress NH_3_ emissions [35].

In this experiment, the proportion of NH_3_ emissions during the SF1 period in the rice season was consistently higher than during the BF and SF2 periods (Figure 2c). Higher temperature during SF1, which not only enhances soil urease activity, accelerating urea hydrolysis, but also leads to a rapid short-term increase in NH_4_^+^ concentration in floodwater, promoting physical diffusion and resulting in greater NH_3_ volatilization losses [36,37]. Therefore, reducing NH_3_ emissions during SF1 is critical for minimizing total NH_3_ losses. NH_3_ flux during BF was generally higher than during SF2 across nearly all treatments (Figure 2c). This difference may be explained by the sparse rice growth during BF, leading to reduced N uptake from the soil and consequently higher N losses via NH_3_ volatilization. In contrast, during SF2, denser plant growth physically obstructs gas exchange, thereby reducing NH_3_ emissions [38]. In the wheat season, HC-OF exhibited the lowest NH_3_ flux during both BF and SF periods, whereas other treatments showed significantly higher NH_3_ flux during BF compared to SF (Figure 2d). This discrepancy may arise because wheat is in the germination stage during BF, with minimal N utilization from the soil, and the higher N application rate during BF contributes to greater NH_3_ losses. In contrast, during SF, the vigorous growth of wheat increases its N demand, resulting in lower NH_3_ flux.

As a key indicator of soil salinization, pH influences significantly N transformation and transport processes in saline–alkali paddy fields [39]. Because BC-OF and HC-OF are alkaline, they readily react with cations in the soil after being applied. Therefore, the application of biochar-amended organic fertilizer can alter the concentration of exchangeable H^+^ and other parameters in the soil, which may subsequently affect the pH of floodwater. The results showed that BC-OF and HC-OF primarily reduced soil NH_3_ compared with CK during the BF stage. Correspondingly, the pH of floodwater under the HC-OF treatment was also relatively lower at the BF stage. For SF1, floodwater pH initially increased, then decreased, followed by a sharp rise on the final day. In contrast, SF2 displayed no significant fluctuations, maintaining a gradual decreasing trend. NH_4_^+^ tends to convert to NH_3_ in saline water with higher pH levels [40]. Therefore, during the early stage of each fertilization period, as the pH of floodwater increases, NH_3_ emissions correspondingly rise. However, the trends of these two parameters diverge in the middle and late stages. Notably, both BF and SF1 demonstrated relatively higher pH values during their respective fertilization periods. This phenomenon may be attributed to the proliferation of green algae in the floodwater during fertilization, where photosynthetic CO_2_ consumption led to pH elevation. Additionally, algal coverage formed a physical barrier that reduced aeration, inhibiting NH_4_^+^-N volatilization. The shading effect also lowered water temperature, slowing urea hydrolysis and NH_4_^+^-N diffusion, thereby reducing NH_3_ emissions. This explains the discrepancy between floodwater pH trends and NH_3_ volatilization patterns.

Previous studies have confirmed that soil NH_3_ flux is closely related to N speciation in floodwater and topsoil [41]. Wang et al. [42] reported a significant positive correlation between NH_4_^+^-N concentration and NH_3_ volatilization in saline–alkali paddy fields. In this study, the NH_4_^+^-N concentration trends across treatments matched NH_3_ volatilization patterns, with SF1 showing a higher proportion of NH_3_ loss, consistent with prior findings. The BC-OF treatment exhibited lower NH_4_^+^-N concentrations in BF compared to other treatments, corresponding to its lowest NH_3_ flux during this stage. This may be attributed to the porous structure of biochar-amended slow-release fertilizer, which effectively adsorbs NH_4_^+^-N and NO_3_^−^-N, thereby reducing N loss. Furthermore, it suppresses organic N mineralization and optimizes N supply dynamics through a controlled-release mechanism [43]. This study found that changes in soil NH_4_^+^-N concentration during the wheat topdressing period were consistent with the corresponding NH_3_ dynamics (Figure 2b), exhibiting a sharp increase in the early fertilization stage followed by a continuous decline in the later stage (Figure 4e). Our results confirm previous research indicating that as soil NH_4_^+^-N increases, both the rate and cumulative loss of NH_3_ volatilization rise sharply [44]. Conversely, NH_3_ emissions decrease progressively as soil NH_4_^+^-N content declines in the later stage. This study has not sufficiently elucidated the mechanisms through which the treatment influences soil microbial communities and crop salt-tolerance physiology, as its focus was on macro-level effects like ammonia reduction. Investigating these mechanisms should be a goal for future work.

### 3.3. Changes in Saline–Alkali Soil Fertilty as Impacted by Different Organic Fertilizer

The reduction in soil pH was relevant to the release of acidic functional groups on the surface of hydrochar (-COOH, -OH) [45], which directly release H^+^ into the soil solution, thereby neutralizing alkaline soil. The hydrochar exhibited a low total acidity of 0.6 mmol/g, dominated by lactonic groups, which is approximately an order of magnitude lower than the acidities of natural humic and fulvic acids reported by Stevenson [46]. This is because the thermochemical conversion process favors the development of stable carbon matrices rather than abundant acidic functional groups. In this study, compared to CK, the HC-OF treatment significantly decreased post-harvest soil pH, corroborating previous findings. When N fertilizers are applied to soil, a substantial portion of N can be enriched and transferred into the soil matrix [47], with a fraction ultimately absorbed by crops [48]. The results indicate that the application of biochar-based organic fertilizers (BC-OF and HC-OF) significantly increased TN and NH_4_^+^-N content compared to CK (Table 2), aligning with prior research [49]. Yu et al. [50] found that hydrochar enhances NH_4_^+^ and NO_3_^−^ retention in soil through electrostatic attraction and pore-filling adsorption mechanisms, leading to gradual N release and reduced leaching. Furthermore, HC-OF exhibits superior N retention capacity due to its abundant oxygen-containing functional groups, which enhance NH_4_^+^ adsorption [51]. The labile organic biochar on the surface of biochar-amended organic fertilizers may be released into the soil upon incorporation, directly increasing SOM content [52]. The results show that HC-OF treatment yielded the highest post-harvest SOM content (Table 3). This could also be attributed to the pH-modulating effect of HC-OF, which may inhibit SOM dissolution in saline–alkali soils, further enhancing SOM retention [53]. The presence of abundant SOM in the soil, coupled with its decomposition, can release excessive organic acids, enhance leaching of base cations, and promote immobilization and denitrification of nitrate [54]. This ultimately shifts the soil to a weakly acidic state [55] and improves saline–alkaline conditions. Although other soil nutrients (NO_3_^−^-N, AP, and AK) did not show statistically significant differences among treatments, BC-OF and HC-OF exhibited slight increases compared to CK. This non-significant but consistent increasing pattern, combined with the significant improvements in pH and TN, collectively suggests that biochar-based organic fertilizers enhance the nutrient retention capacity of saline–alkali soils through physical adsorption and chemical regulation, supporting their potential for soil reclamation and sustainable utilization.

## 4. Materials and Methods

### 4.1. Experimental Setup

#### 4.1.1. Experimental Materials

The pot experiment was conducted from June 2023 to May 2024 in a greenhouse of Jiangsu Academy of Agricultural Sciences (32°08′ N, 118°82′ E) in an East Asian monsoon climate zone with an annual precipitation of 1106.5 mm and an average annual temperature of 15.5 °C. The soil used was saline–alkali soil, with the following basic physicochemical properties: pH 8.54, EC 0.99 Ds/m, SOM 8.93 g/kg, TN 0.61 g/kg, AP 19.84 mg/kg, AK 145.47 mg/kg, and salt content 14.4‰. Soil columns were constructed from PVC material, with a height of 60 cm and a diameter of 30 cm. Fertilizers comprised urea (46% N), calcium superphosphate (46% P_2_O_5_), and potassium chloride (60% K_2_O), which were all commercially sourced.

Three organic fertilizers, i.e., conventional organic fertilizer (OF), biochar-amended organic fertilizer (BC-OF), and hydrochar-amended organic fertilizer (HC-OF) were applied. OF is primarily prepared using chicken manure, spent mushroom substrate, and fermentation materials as the main raw ingredients.

BC derived from wheat straw in a continuous slow pyrolysis system at 500 °C was evaluated in this experiment. The reactor (SGM·M12, Sigma, China) was heated by a stepwise procedure under oxygen-limited conditions. In detail, when pyrolyzed, the temperature was raised to 500 °C, at a rate of 5 °C min^−1^ and held for 8 h, and then the temperature was decreased to room temperature at a rate of 5 °C min^−1^ [56]. HC was synthesized via hydrothermal carbonization: wheat straw and water were mixed at a 1:10 mass ratio and reacted in a high-pressure reactor (BR-10L, China) at 260 °C for 1 h. The solid product was collected after solid–liquid separation and drying [57]. The mixing ratio in biochar and hydrochar with organic fertilizer is 15%, which was amended primarily based on the existing literature [58]. The number of surface acidic functional groups on the hydrochar was determined by Boehm titration according to Zhang et al. [59] and were carboxylic 0.09 mmol/g, lactonic 0.46 mmol/g, phenolic 0.05 mmol/g, and total acidity 0.60 mmol/g.

#### 4.1.2. Experimental Design and Management

This study employed a rice–wheat rotation system initiated during the 2023 rice season with four treatments: (1) CK (urea only, 200 kg/hm^2^); (2) OF (urea + organic fertilizer); (3) BC-OF (urea + biochar-amended organic fertilizer); and (4) HC-OF (urea + hydrochar-amended organic fertilizer), each replicated four times. Organic fertilizers were incorporated at 1% (*w*/*w*) of topsoil (10 kg). Calculated on the basis of the cross-sectional area of the experimental soil column, this amounts to 14.15 t/ha. The fertilizer input per pot was adjusted based on the farmers’ fertilization experience and the field application rate.

The experiment was conducted in a conventional water and fertilizer management mode, with N fertilizer supplied by urea (46% N content) in the ratio of 30%:30%:40% as basal, tiller, and spike fertilizers during the rice season. Both P and K fertilizers were applied as basal fertilizers in the forms of calcium superphosphate and potassium chloride at the rates of 96 kg/ha P_2_O_5_ and 192 kg/ha K_2_O as a single application. The tested rice (*Oryza sativa* L., var. Nangeng 46) was transplanted in June 2023, with three holes per soil column and three seedlings per hole, and was harvested in October. The water level was kept at 3–5 cm during the growing period of rice and the medium drainage was carried out from 18 to 28 July 2023. In the wheat (*Triticum aestivum* L. var. Ningmai 26) season, the total N application was maintained at 120 kg/ha, split between basal fertilization (60% N) and topdressing (40% N). All other fertilization practices remained consistent with the rice season. Wheat was sown in December 2023 at a density of 65 seeds per pot, thinned to 30 uniform seedlings, and harvested in May 2024.

### 4.2. Sample Collection and Analysis

#### 4.2.1. Yield and N Use Efficiency (NUE)

At maturity, straw and grains were separately harvested, with panicle number per soil column, grain number per panicle, and fresh weights recorded. Dried straw and grain samples were pretreated (Grinder FW-100, Beijing Guangming Medical Apparatus Co., Ltd., Beijing, China), digested by H_2_SO_4_-H_2_O_2_ oxidation, and analyzed for TN content via the Kjeldahl method (Distillation Apparatus, KD200, Jiasheng Technology Co., Ltd., Anhui, China). N uptake (straw and grains) and NUE were subsequently calculated:

(1) $$\mathrm{Straw}\;\left(\mathrm{grain}\right)\;N\;\mathrm{uptake}\left(g\right)\;=\;\mathrm{dry}\;\mathrm{weight}\;\mathrm{of}\;\mathrm{straw}\left(\mathrm{grain}\right)\;\left(g\right)\;\times \;N\;\mathrm{content}\;\mathrm{of}\;\mathrm{straw}\;(\mathrm{grain})\;(g/\mathrm{kg})/1000$$

(2) $$N\;\mathrm{use}\;\mathrm{efficiency}\;\left(\%\right)=\frac{\;N\;\mathrm{uptake}\;\mathrm{in}\;N\;\mathrm{application}\;\mathrm{treatment}\;\left(g\right)-N\;\mathrm{uptake}\;\mathrm{in}\;\mathrm{control}\;\left(g\right)}{N\;\mathrm{application}\;\mathrm{rate}\;\left(g\right)}\;\times \;100\%$$

#### 4.2.2. Floodwater pH, NH_4_^+^-N, and NO_3_^−^-N Concentration

In the rice-growing season, approximately 50 mL of floodwater was collected from paddy fields on Days 1, 3, 5, and 7 after fertilization at BF, SF1, and SF2 stages. The samples were immediately transported to the laboratory and frozen. The pH of the floodwater was measured in situ using a portable pH meter (Leici E-301-QC, Shanghai Yidian Scientific Instrument Co., Ltd., Shanghai, China). NH_4_^+^-N and NO_3_^−^-N concentrations were determined by the indophenol blue colorimetric method and ultraviolet spectrophotometry (T-6M, Nanjing Feile Instrument Co., Ltd., Nanjing, China), respectively.

#### 4.2.3. NH_3_ Volatilization

NH_3_ volatilization was quantified using a continuous air-flow enclosure method following Feng et al. [56]. The absorption bottles (15 cm inner diameter × 20 cm height) were filled with 80 mL of a 2% (*w*/*v*) boric acid (H_3_BO_3_) trapping solution, supplemented with 20 mL of methyl red-bromocresol green-ethanol indicator mixture per liter of absorbent. The trapped NH_3_ was immediately titrated post-collection using 0.02 mol/L H_2_SO_4_ to determine daily volatilization rates. Cumulative NH_3_ emission was calculated as the sum of daily measurements throughout the experimental period:

(3) $${\mathrm{NH}}_{3}\;\left(\mathrm{kg}/{\mathrm{hm}}^{2}/d\right)\;=\;V\;\times \;10^{-3}\;\times \;C\;\times \;0.014\;\times \;10^{4}/\pi \;\times \;r^{2}\;\times \;6$$

In the formula: V is the volume of sulfuric acid used for titration (mL); 10^−3^ is the volume conversion factor; C is the calibrated concentration of H_2_SO_4_ used for titration (mol/L); 0.014 is the relative atomic mass of N (kg/mol); 10^4^ is the area conversion factor; r is the radius of the chamber (m); and 6 is the ratio of 24 h to the daily NH_3_ volatilization collection time of 4 h.

#### 4.2.4. Soil Sampling and Analysis

Soil samples (0–20 cm depth) were collected after rice harvest, on Days 4 and 12 following each wheat fertilization stage and after wheat harvest. The samples were air-dried, sieved (2 mm and 0.15 mm mesh sizes), and manually cleaned of crop residues. Fresh subsamples were stored frozen in a laboratory freezer for subsequent soil property measurements. Soil pH was determined potentiometrically using a pH electrode (soil/water ratio = 1:2.5). The electrical conductivity (EC) of the soil is measured using a conductivity meter (DDSJ-308F, Shanghai Yidian Scientific Instrument Co., Ltd., Shanghai, China). SOM and TN were analyzed via the K_2_Cr_2_O_7_-H_2_SO_4_ oxidation method and the Kjeldahl method (Distillation Apparatus, KD200, Jiasheng Technology Co., Ltd., Anhui, China), respectively. NH_4_^+^-N and NO_3_^−^-N concentrations were measured using the indophenol blue colorimetric method and UV spectrophotometry, respectively. AP was extracted with 0.5 mol/L NaHCO_3_ and quantified via the molybdenum-antimony anti-colorimetric method (T-6M, Nanjing Feile Instrument Co., Ltd., Nangjing, China), while AK was extracted with 1 mol/L NH_4_OAc and analyzed by flame photometry (FP6410, Shanghai Yidian Scientific Instrument Co., Ltd., Shanghai, China).

### 4.3. Statistical Analysis

The data were collated and analyzed by ANOVA using Excel 2010 and SPSS 26.0 software (SPSS Inc., Chicago, IL, USA). Multiple comparison tests were performed between the different treatments using Duncan’s method, with different lowercase letters indicating significant differences between treatments at the significance level of *p* < 0.05.

## 5. Conclusions

A pot experiment was conducted to investigate the effects of OF, BC-OF, and HC-OF on crop yield, soil nutrient characteristics, and NH_3_ volatilization in saline–alkali soil with a rice–wheat rotation. The results showed that OF, BC-OF, and HC-OF applications increased rice and wheat grain yields, with HC-OF exhibiting the highest yield enhancement. The HC-OF treatment significantly reduced soil pH, while BC-OF and HC-OF treatments significantly increased TN and NH_4_^+^-N contents. However, there is no significant change in soil EC, NO_3_^−^-N, AP, AK, and SOM contents after three types of organic fertilizer application. Compared to the CK treatment, the applications of OF, BC-OF, and HC-OF effectively mitigated NH_3_ emissions from the rice–wheat rotation soil system. Of which, HC-OF exerted the highest efficacy in mitigating NH_3_ volatilization, in particular that from the wheat season. In summary, compared with BC-OF, HC-OF is better in managing saline–alkali soils as it could enhance crop yield while reducing NH_3_ loss. Meanwhile, the long-term effects of varied organic fertilizers on N losses, crop production, and fertilities of saline–alkali soils require further investigation.

## Figures and Tables

**Table 1:** Effects of organic fertilizer (OF) and biochar- (BC-OF) and hydrochar-amended organic fertilizer (HC-OF) application on yield and agronomic traits of rice and wheat.

Crop Season	Treatment	Grain Yield (g/pot)	Spike Number	Kernels per Spike	Thousand-Kernel Weight (g)	Annual Yield
Rice	CK	77.29 ± 1.81 a	28.50 ± 1.12 a	105.98 ± 5.35 a	26.62 ± 0.59 a	110.13 ± 2.01 a
	OF	82.97 ± 4.48 a	31.25 ± 1.47 a	104.46 ± 4.77 a	25.38 ± 0.60 a	113.24 ± 4.31 a
	BC-OF	86.17 ± 4.88 a	30.75 ± 1.30 a	109.44 ± 3.73 a	25.75 ± 1.14 a	118.26 ± 1.39 a
	HC-OF	87.18 ± 3.17 a	30.75 ± 2.17 a	115.53 ± 4.94 a	26.27 ± 0.63 a	122.45 ± 4.52 a
Wheat	CK	29.73 ± 1.29 b	35.25 ± 2.68 a	20.57 ± 1.79 a	41.25 ± 1.28 a	
	OF	30.26 ± 0.76 b	35.50 ± 2.69 a	22.20 ± 2.23 a	38.76 ± 1.58 a	
	BC-OF	32.09 ± 1.26 ab	35.25 ± 2.68 a	22.72 ± 1.87 a	40.36 ± 2.33 a	
	HC-OF	35.27 ± 1.46 a	38.50 ± 2.60 a	23.69 ± 1.66 a	38.77 ± 0.92 a	

Note: The values are means ± SD (*n* = 4). Different letters mean statistically significant differences at *p* < 0.05.

**Table 2:** Post-harvest soil physicochemical properties of organic fertilizer (OF) and biochar- (BC-OF) and hydrochar-amended organic fertilizer (HC-OF) treatments.

Treatment	pH	EC (μs/cm)	TN (g/kg)	NH_4_^+^-N (mg/kg)	NO_3_^−^-N (mg/kg)	AP (mg/kg)	AK (mg/kg)	SOM (g/kg)
CK	7.88 ± 0.05 a	959 ± 37.50 a	0.53 ± 0.01 c	1.43 ± 0.45 b	28.30 ± 3.14 a	5.32 ± 0.27 a	243.20 ± 3.47 a	9.27 ± 0.80 a
OF	7.78 ± 0.05 ab	1077.50 ± 11.50 a	0.55 ± 0.02 bc	0.47 ± 0.12 b	30.04 ± 2.51 a	5.68 ± 0.43 a	283.67 ± 2.89 a	9.40 ± 0.86 a
BC-OF	7.80 ± 0.08 ab	926.25 ± 25.00 a	0.57 ± 0.03 ab	4.70 ± 0.08 a	31.86 ± 2.50 a	5.43 ± 0.51 a	270.66 ± 5.10 a	9.11 ± 0.32 a
HC-OF	7.75 ± 0.08 b	1185.25 ± 14.50 a	0.59 ± 0.01 a	4.14 ± 0.25 a	35.25 ± 1.46 a	5.32 ± 0.87 a	259.86 ± 7.65 a	10.30 ± 0.51 a

Note: The values are means ± SD (*n* = 4). Different letters mean statistically significant differences at *p* < 0.05.

**Table 3:** Detailed characteristics of organic fertilizer (OF) and biochar- (BC-OF) and hydrochar-amended organic fertilizer (HC-OF) used in the current experiment.

Treatment	pH	EC (ms/cm)	TN (g/kg)	AP (mg/kg)	AK (g/kg)	OM (g/kg)
OF	7.29	3.75	19.28	69.96	1.13	427.28
BC-OF	8.34	3.90	19.29	28.98	1.99	419.67
HC-OF	7.62	4.18	23.21	113.96	1.27	439.45

## Data Availability

The original contributions presented in this study are included in the article. Further inquiries can be directed to the corresponding author.

## References

[1] Skrzypek G., Dogramaci S., Rouillard A., Grierson P.F. (2016). Groundwater seepage controls salinity in a hydrologically terminal basin of semi-arid northwest Australia. J. Hydrol..

[2] Hopmans J.W., Qureshi A.S., Kisekka I., Munns R., Grattan S.R., Rengasamy P., Taleisnik E. (2021). Critical knowledge gaps and research priorities in global soil salinity. Adv. Agron..

[3] Li-ping L., Xiao-hua L., Hong-bo S., Zhao-Pu L., Ya T., Quan-suo Z., Jun-qin Z. (2015). Ameliorants improve saline–alkaline soils on a large scale in northern Jiangsu Province, China. Ecol. Eng..

[4] Hassani A., Azapagic A., Shokri N. (2020). Predicting long-term dynamics of soil salinity and sodicity on a global scale. Proc. Natl. Acad. Sci. USA.

[5] Yu S., Li J., Yingxin L., Hongbin W., Deliang L., Chunjie T. (2024). Compositional and functional succession of soil bacterial communities during long-term rice cultivation on saline-alkali soils: Insights derived from a new perspective of the core bacterial taxa. Pedosphere.

[6] Sahab S., Suhani I., Srivastava V., Chauhan P.S., Singh R.P., Prasad V. (2021). Potential risk assessment of soil salinity to agroecosystem sustainability: Current status and management strategies. Sci. Total Environ..

[7] Liu M., Wang C., Liu X., Lu Y., Wang Y. (2020). Saline-alkali soil applied with vermicompost and humic acid fertilizer improved macroaggregate microstructure to enhance salt leaching and inhibit nitrogen losses. Appl. Soil Ecol..

[8] Ren T., Fan P., Zuo W., Liao Z., Wang F., Wei Y., Liu G. (2023). Biochar-amended fertilizer under drip irrigation: More conducive to improving soil carbon pool and promoting nitrogen utilization. Ecol. Indic..

[9] Rasool M., Akhter A., Soja G., Haider M.S. (2021). Role of biochar, compost and plant growth promoting rhizobacteria in the management of tomato early blight disease. Sci. Rep..

[10] Feng H.L., Xu C.S., He H.H., Zeng Q., Liu G.S. (2021). Effect of biochar on soil enzyme activity & the bacterial community and its mechanism. Environ. Sci..

[11] Chivenge P., Vanlauwe B., Gentile R., Wangechi H., Mugendi D., Van Kessel C., Six J. (2009). Organic and mineral input management to enhance crop productivity in Central Kenya. Agron. J..

[12] Ibrahim A., Abaidoo R.C., Fatondji D., Opoku A. (2015). Hill placement of manure and fertilizer micro-dosing improves yield and water use efficiency in the Sahelian low input millet-based cropping system. Field Crops Res..

[13] Bento L.R., Castro A.J.R., Moreira A.B., Ferreira O.P., Bisinoti M.C., Melo C.A. (2019). Release of nutrients and organic carbon in different soil types from hydrochar obtained using sugarcane bagasse and vinasse. Geoderma.

[14] Bona D., Bertoldi D., Borgonovo G., Mazzini S., Ravasi S., Silvestri S., Tambone F. (2023). Evaluating the potential of hydrochar as a soil amendment. Waste Manag..

[15] Khosravi A., Zheng H., Liu Q., Hashemi M., Tang Y., Xing B. (2022). Production and characterization of hydrochars and their application in soil improvement and environmental remediation. Chem. Eng. J..

[16] He K., Xu Y., He G., Zhao X., Wang C., Li S., Hu R. (2023). Combined application of acidic biochar and fertilizer synergistically enhances Miscanthus productivity in coastal saline-alkaline soil. Sci. Total Environ..

[17] Mustafa A., Hu X., Abrar M.M., Shah S.A.A., Nan S., Saeed Q., Minggang X. (2021). Long-term fertilization enhanced carbon mineralization and maize biomass through physical protection of organic carbon in fractions under continuous maize cropping. Appl. Soil Ecol..

[18] Ju X.T., Kou C.L., Christie P., Dou Z.X., Zhang F.S. (2007). Changes in the soil environment from excessive application of fertilizers and manures to two contrasting intensive cropping systems on the North China Plain. Environ. Pollut..

[19] Bodirsky B.L., Popp A., Lotze-Campen H., Dietrich J.P., Rolinski S., Weindl I., Stevanovic M. (2014). Reactive nitrogen requirements to feed the world in 2050 and potential to mitigate nitrogen pollution. Nat. Commun..

[20] Akhtar M., Hussain F., Ashraf M.Y., Qureshi T.M., Akhter J., Awan A.R. (2012). Influence of Salinity on Nitrogen Transformations in Soil. Commun. Soil Sci. Plant Anal..

[21] Feng Y., He H., Li D., He S., Yang B., Xue L., Chu Q. (2021). Biowaste hydrothermal carbonization aqueous product application in rice paddy: Focus on rice growth and ammonia volatilization. Chemosphere.

[22] Zheng H., Wang Z., Deng X., Herbert S., Xing B. (2013). Impacts of adding biochar on nitrogen retention and bioavailability in agricultural soil. Geoderma.

[23] Dong Y., Zhang X., Wang X., Xie C., Liu J., Cheng Y., Li Y. (2024). Modified biochar affects CO_2_ and N_2_O emissions from coastal saline soil by altering soil pH and elemental stoichiometry. Sci. Total Environ..

[24] Wang C., Sun H., Zhang X., Zhang J., Zhou S. (2023). Optimal straw retention strategies for low-carbon rice production: 5 year results of an in situ trial in eastern China. Agronomy.

[25] Liu X., Zhou J., Chi Z., Zheng J., Li L., Zhang X., Pan G. (2019). Biochar provided limited benefits for rice yield and greenhouse gas mitigation six years following an amendment in a fertile rice paddy. Catena.

[26] Huang M., Fan L., Chen J., Jiang L., Zou Y. (2018). Continuous applications of biochar to rice: Effects on nitrogen uptake and utilization. Sci. Rep..

[27] Chen M., Zhang S., Liu L., Wu L., Ding X. (2021). Combined organic amendments and mineral fertilizer application increase rice yield by improving soil structure, P availability and root growth in saline-alkaline soil. Soil Tillage Res..

[28] Selvarajh G., Ch’ng H.Y. (2021). Enhancing Soil Nitrogen Availability and Rice Growth by Using Urea Fertilizer Amended with Rice Straw Biochar. Agronomy.

[29] Selvarajh G., Ch’ng H.Y., Zain N.M., Wei L.S., Liew J.Y., Azmin S.N.H.M., Damrongrak I. (2024). Enriched rice husk biochar superior to commercial biochar in ameliorating ammonia loss from urea fertilizer and improving plant uptake. Heliyon.

[30] Chew J., Joseph S., Chen G., Zhang Y., Zhu L., Liu M., Fan X. (2022). Biochar-based fertiliser enhances nutrient uptake and transport in rice seedlings. Sci. Total Environ..

[31] Li Y., Cheng J., Lee X., Chen Y., Gao W., Pan W., Tang Y. (2019). Effects of biochar-based fertilizers on nutrient leaching in a tobacco-planting soil. Acta Geochim..

[32] Shi Z.Q., She D.L., Chen X.Y., Xia Y.Q. (2021). Effects of salinity on soil ammonia volatilization and denitrification rates. J. Ecol. Rural Environ..

[33] Shuai H., Guodong W., Lei Z. (2023). The Effect of Interaction between Biochar and Nitrogen Fertilizer on Ammonia Volatilization in Salinized Soil. J. Irrig. Drain..

[34] Kastner J.R., Miller J., Das K.C. (2009). Pyrolysis conditions and ozone oxidation effects on ammonia adsorption in biomass generated chars. J. Hazard. Mater..

[35] Zhang S., Zhu X., Zhou S., Shang H., Luo J., Tsang D.C. (2019). Hydrothermal carbonization for hydrochar production and its application. Biochar Biomass Waste.

[36] Xie W.M., Li S.J., Shi W.M., Zhang H.L., Fang F., Wang G.X., Zhang L.M. (2020). Quantitatively ranking the influencing factors of ammonia volatilization from paddy soils by grey relational entropy. Environ. Sci. Pollut Res..

[37] Wang B., Li R., Wan Y., Li Y.E., Cai W., Guo C., Wilkes A. (2021). Air warming and CO_2_ enrichment cause more ammonia volatilization from rice paddies: An OTC field study. Sci. Total Environ..

[38] Abulaiti A., She D., Zhang W., Xia Y. (2023). Regulation of denitrification/ammonia volatilization by periphyton in paddy fields and its promise in rice yield promotion. J. Sci. Food Agric..

[39] Chi C.M., Zhao C.W., Sun X., Wang Z.C. (2012). Reclamation of saline-sodic soil properties and improvement of rice (*Oriza sativa* L.) growth and yield using desulfurized gypsum in the west of Songnen Plain, northeast China. Geoderma.

[40] Francis D.D., Vigil M.F., Mosier A.R. (2008). Gaseous losses of nitrogen other than through denitrification. Nitrogen Agric. Syst..

[41] Feng Y., Han L., Li D., Sun M., Wang X., Xue L., Xing B. (2022). Presence of microplastics alone and co-existence with hydrochar unexpectedly mitigate ammonia volatilization from rice paddy soil and affect structure of soil microbiome. J. Hazard. Mater..

[42] Wang X., Wang M., Chen L., Shutes B., Yan B., Zhang F., Zhu H. (2023). Nitrogen migration and transformation in a saline-alkali paddy ecosystem with application of different nitrogen fertilizers. Env. Sci. Pollut Res..

[43] Bakshi S., Banik C., Laird D.A., Smith R., Brown R.C. (2021). Enhancing biochar as scaffolding for slow release of nitrogen fertilizer. ACS Sustain. Chem. Eng..

[44] Rochette P., Angers D.A., Chantigny M.H., Gasser M.O., MacDonald J.D., Pelster D.E., Bertrand N. (2013). NH3 volatilization, soil concentration and soil pH following subsurface banding of urea at increasing rates. Can. J. Soil Sci..

[45] Rehman M.Z., Batool Z., Ayub M.A., Hussaini K.M., Murtaza G., Usman M., Ali S. (2020). Effect of acidified biochar on bioaccumulation of cadmium (Cd) and rice growth in contaminated soil. Environ. Technol. Innov..

[46] Stevenson F.J. (1994). Genesis, composition, and reactions. Humus Chemistry.

[47] Feng Y., Du H., Wulandari T., Poinern G.E.J., Jiang Z.T., Fawcett D., Yang L. (2022). Hydrochar amendments stimulate soil nitrous oxide emission by increasing production of hydroxyl radicals and shifting nitrogen functional genes in the short term: A culture experiment. Chemosphere.

[48] de la Rosa J.M., Paneque M., Miller A.Z., Knicker H. (2014). Relating physical and chemical properties of four different biochars and their application rate to biomass production of Lolium perenne on a Calcic Cambisol during a pot experiment of 79 days. Sci. Total Environ..

[49] Melo T.M., Bottlinger M., Schulz E., Leandro W.M., de Aguiar Filho A.M., Wang H., Rinklebe J. (2018). Plant and soil responses to hydrothermally converted sewage sludge (sewchar). Chemosphere.

[50] Yu S., Feng Y., Xue L., Sun H., Han L., Yang L., Chu Q. (2019). Biowaste to treasure: Application of microbial-aged hydrochar in rice paddy could improve nitrogen use efficiency and rice grain free amino acids. J. Clean. Prod..

[51] Chu Q., Xue L., Singh B.P., Yu S., Müller K., Wang H., Yang L. (2020). Sewage sludge-derived hydrochar that inhibits ammonia volatilization, improves soil nitrogen retention and rice nitrogen utilization. Chemosphere.

[52] Liu K., Ran Q., Li F., Shaheen S.M., Wang H., Rinklebe J., Fang L. (2022). Carbon-based strategy enables sustainable remediation of paddy soils in harmony with carbon neutrality. Carbon Res..

[53] Yuan Y., Liu Q., Zheng H., Li M., Liu Y., Wang X., Xing B. (2023). Biochar as a sustainable tool for improving the health of salt-affected soils. Soil Environ. Health.

[54] Liang F., Li B., Vogt R.D., Mulder J., Song H., Chen J., Guo J. (2023). Straw return exacerbates soil acidification in major Chinese croplands. Resour. Conserv. Recycl..

[55] Zhao W., Hu W., Zhang F., Shi Y., Wang Y., Zhang X., Xu R. (2024). Exchangeable acidity characteristics of farmland black soil in northeast China. Geoderma Reg..

[56] Feng Y., Sun H., Xue L., Liu Y., Gao Q., Lu K., Yang L. (2017). Biochar applied at an appropriate rate can avoid increasing NH3 volatilization dramatically in rice paddy soil. Chemosphere.

[57] Feng Y., Tang Q., Xie W., Yu J., Wang L., Wang B. (2025). Application of products derived from pyrolysis and hydrothermal carbonization as conditioners for aerobic composting produced multiple beneficial effects: Evaluation amended on 10-ton pilot scale trials. Chem. Eng. J..

[58] Huang W., Sun H., Sun X., Gong X., Bian R., Wang Y., Feng Y. (2024). Co-amendment of dicyandiamide with waste carbonization products into composting: Enhanced fertility, reduced gas emission and increased economic benefits. J. Clean. Prod..

[59] Zhang W., Han B., Wille U., Butterly C., He J., Chen D. (2022). Surface modification of coal tailings by thermal air oxidation for ammonia capture. J. Clean. Prod..

